# Genetic Variability in Child Growth Among South American Populations: A Perspective Integrating Population Genetics, Growth Standards, and Precision Growth Medicine

**DOI:** 10.3390/ijms26199300

**Published:** 2025-09-23

**Authors:** Ana Karina Zambrano, Patricia Guevara-Ramírez, Santiago Cadena-Ullauri, Carmen Basantes, Susana Nicola, Susana Hidalgo, Maria L. Felix

**Affiliations:** 1 Universidad UTE, Facultad de Ciencias de la Salud Eugenio Espejo, Centro de Investigación Genética y Genómica, Quito 170129, Pichincha, Ecuador; 2 Universidad UTE, Facultad de Ciencias de la Salud Eugenio Espejo, Quito 170129, Pichincha, Ecuador; 3 Universidad UTE, Centro de Investigación en Salud Pública y Epidemiología Clínica, Facultad de Ciencias de la Salud Eugenio Espejo, Quito 170129, Pichincha, Ecuador

**Keywords:** healthcare, growth, height, South America, genetics, Native American

## Abstract

Child growth in South America results from a complex interplay of genetic, environmental, and socioeconomic factors. The region’s high ancestral diversity—stemming from Native American, European, and African admixture—shapes growth patterns in ways not fully captured by international standard curves such as World Health Organization (WHO) charts, which are primarily based on European population. This mismatch may cause misclassification, especially among Native American and other underrepresented groups, and reduce the effectiveness of interventions like growth hormone (GH) therapy. Evidence from national surveys, cohort studies, and genetic analyses reveals persistent ethnic and socioeconomic disparities, with Native American children showing higher stunting prevalence even after adjusting for wealth and residence. Differences between WHO and national growth curves further contribute to inconsistent prevalence estimates due to methodological and contextual variants. Regional genomic studies, although limited, have identified population-specific variants, such as *FBN1* (E1297G) in Peru, and modulators of GH therapy response, including *GHR* exon 3 deletion, *ACAN*, and *NPR2*, highlighting the role of genetic background, treatment timing, and adherence in height outcomes. These findings underscore the need to move toward precision growth medicine, integrating anthropometry, genetic, environmental, and socioeconomic data to design population-specific growth references, optimize pharmacogenetic approaches, and reduce inequities in pediatric growth care.

## 1. Introduction

Growth is a biological process resulting from the complex interaction of exogenous factors—nutritional, physical activity, environmental, economic, social, and cultural factors—and endogenous factors—genetic, hormonal, and metabolic factors, as well as the tissue response of plate cartilage growth—which determine a unique growth velocity and pattern for each individual throughout the fetal, childhood, and adolescent stages [1,2].

Critical growth periods, such as the first 1000 days of life, are particularly important due to the programming effects of specific nutritional and metabolic conditions on future health during adolescence and adulthood. These conditions have long-term implications for body composition, brain development, gut microbiota characteristics, and immune system maturation [3,4].

Globally, growth assessment from gestation through adolescence is based on anthropometric measurements compared with reference growth curves or tables considered to represent optimal growth, such as longitudinal reference curves [5,6] and cross-sectional curves applicable to specific populations [7,8]. These tools are essential for the timely detection and management of conditions that alter growth patterns and impact physical, emotional, and cognitive development in both the short and long term [9].

Applying universal growth standards, designed from specific populations, poses challenges when transferred without adjustment to regional contexts with high ethnic, environmental, and socioeconomic diversity, as in South America [8,10]. Evidence indicates that global standards—such as those of the World Health Organization (WHO)—may lead to over- or underdiagnoses of nutritional problems, introducing bias into clinical practice and epidemiological surveillance [8,10].

An even greater challenge is the incorporation of genetic analysis, which can differentiate normal growth variants—such as constitutional delay and familial short stature—from genetic syndromes or mutations affecting growth hormone or its receptor. This distinction is crucial for determining the appropriateness of growth hormone therapy [11,12]. In South America, the high genetic variability results from a complex admixture of Native American, European, and African ancestries [13,14].

The most common treatment is growth hormone administration, used for managing short stature in Turner syndrome, Prader–Willi syndrome, Noonan syndrome, growth hormone deficiency, small-for-gestational-age children without catch-up growth by two years of age, *SHOX* (Short Stature Homeobox) gene deletions/mutations, chronic kidney disease, and idiopathic short stature [15].

Studies in South America have reported a 74.7% effectiveness rate in patients with growth hormone deficiency, with an estimated cost of USD 8860 per patient in 2019 [16]. These costs must be considered within the context of a region where infant morbidity and mortality are closely linked to inequities in healthcare access, poor sanitation, inadequate governmental policies, unequal distribution of health resources, and socioeconomic vulnerabilities, particularly among adolescent mothers [17].

The present study aims to describe the genetic variants, biological pathways, socioeconomical context, available growth standard curves, and growth hormone therapy response associated with growth in South American populations.

## 2. The Background of Population Genetics in South America

The history of South America has been shaped by a complex sequence of events, including ancient human migration, colonization, and forced displacement. The initial peopling of the continent occurred approximately 17,000 to 13,000 years ago, when groups originating from Northeast Asia crossed the Bering Strait and gradually dispersed, giving rise to distinct North American and Central and South American populations [18,19]. Over time, these populations adapted to diverse environmental conditions, such as high-altitude living [20], and regions with elevated arsenic concentrations in the soil [21]. These pressures contributed to unique patterns of genetic diversity and population structure across the continent [18,22,23].

These early inhabitants eventually founded the pre-Columbian civilizations, including the Incas, Mayas, and Aztecs [24]. A major turning point in South American history occurred with the arrival of Spanish and Portuguese colonizers in the 15th and 16th centuries. Their expansion across the continent led to demographic collapse among Native American populations due to warfare, and systemic oppression, including forced labor and enslavement [18,25,26]. The transatlantic slave trade further contributed to the region’s demographic changes, as large numbers of African individuals were forcibly brought to the Americas. These interactions among Native American, European, and African groups gave rise to the complex and heterogeneous genetic landscape that characterizes present-day South American populations [18,26]. For instance, although many populations in the region are admixed with components from all three ancestries, the proportion of each varies geographically. Caribbean populations tend to have a higher African component, while Central and Andean regions show a greater Native American ancestry, and southern regions of the continent display a higher proportion of European ancestry [13,19,27,28,29,30,31,32].

Genetic ancestry can be studied using various molecular markers. Mitochondrial DNA (mtDNA), which is maternally inherited and does not undergo recombination, has been widely used to infer maternal ancestry [33,34]. Similarly, the non-recombining portion of the Y chromosome (NRY) provides insights into paternal lineage and has been used extensively for ancestry determination [35,36,37]. Another valuable tool is the use of Short Tandem Repeats (STRs), which are highly polymorphic and commonly applied in forensic genetics for purposes such as identifying missing persons and inferring population structure [18,38,39].

In addition to warfare and slavery, the Native American population of the Americas was drastically reduced by the introduction of infectious diseases such as measles, mumps, smallpox, and influenza, which were previously unknown in the region [18]. These historical processes continue to affect health outcomes today. In particular, individuals with predominantly Native American or African ancestry may face health disparities, including unequal access to medical care and underrepresentation in scientific and genomic research [18]. Even studies conducted in Latin America seem often disproportionately focus on individuals of primarily European ancestry [18]. It is therefore essential to promote the inclusion of Native American and African-descendant individuals in health and genetic research.

In recent years, important efforts have been made to increase South American representation in genomic studies. For instance, the Jaguar Project explores how the genomic structure of the region influences gene expression and immune system regulation [40], The 1000 Genomes Project has also contributed to the characterization of human genetic variation by including individuals from diverse populations around the world, including Latin America [41,42]. More recently, the Genetics of Latin American Diversity (GLAD) Project, launched in 2024, includes data from 53,738 individuals from 46 geographic regions, with the goal of advancing equitable and inclusive genomic research in Latin America [14].

The genetic and demographic history of South America has been shaped by migration and colonization, resulting in a richly admixed and regionally diverse population. However, despite this complex ancestral background, Native American populations remain markedly underrepresented in genetic and genomic research. This lack of inclusion not only limits our understanding of human genetic diversity but also perpetuates health disparities by restricting the applicability of biomedical findings to all population groups.

To promote equitable health outcomes, it is essential to promote the inclusion of Native American individuals in research efforts. Doing so will enhance the resolution of population-specific genetic variation, facilitate the development of ancestry-informed medical interventions, and contribute to a more accurate and comprehensive understanding of human biology. Future genomic initiatives should adopt inclusive frameworks that recognize and respect the historical, cultural, and biological significance of Native American populations in South America.

## 3. The Genetic Landscape of Child Growth and Its Implications for Precision Growth Medicine

### 3.1. Polygenic Regulation of Height

Human height is a highly polygenic trait influenced by additive effects of thousands of genetic variants. Height heritability is high but differs by sex [43]. Studies have measured the heritability of height, reporting ranges in twins, in men it was reported from 0.87 to 0.93, while in women it is lower from 0.68 to 0.84 both in a model of additive genes and unique environment [44].

Over the past two decades, genome-wide association studies (GWASs) have revolutionized our understanding of how genetic variation contributes to human height [45]. One of the earliest GWAS linked the *HMGA2* gene to height, identifying the variant rs1042725, which explained 0.3% of height variation in the population [46]. This study paved the way for subsequent large-scale studies.

Subsequent GWASs included 183,727 individuals reported 180 loci related to height [47]. Another GWAS with data from 253,288 individuals, revealed 697 variants spread across in 423 loci associated with adult height [48]. Later, Yengo et al. (2018) analyzed the data of approximately 250,000 individuals of European ancestry and discovered 3290 single nucleotide polymorphisms (SNPs) associated with height [49]. The most recent GWAS effort, involving 5.4 million people of diverse ancestries, reported 12,111 independent SNPs in 462 autosomal genes linked with height [50].

These studies confirm that while 80–90% of height variation in European ancestry is attributed to genetic factors, much of the remaining variability is attributable to rare variants, gene interactions, and environmental factors such as nutrition, socioeconomic conditions and lifestyle [50]. These studies are crucial for clinical contexts, where distinguishing between a benign polygenic short stature and pathological growth disorders can be challenging.

However, it is important to note that the majority of the GWASs have been conducted in European population, which limits the generalizability of the variants found in other ancestral groups. For example, individuals of Hispanic ethnicity represented only 8.5% of the largest GWAS sample [50]. This underrepresentation highlights the need of regionally representative studies that consider the admixed population such as those in South America, where Native American, European and African ancestry components are distributed.

### 3.2. Key Genes and Biological Pathways Influencing Stature

GWASs have facilitated the identification of numerous genes and genetic variants associated with human height (Table 1). The largest study to date identified 462 autosomal genes linked to stature regulation [50]. These genes are involved in various growth-related signaling pathways, such as:

○ Fibroblast growth factor (FGF) signaling pathway

The fibroblast growth factor (*FGF*) family includes 22 members grouped into six subfamilies, with roles in cell proliferation, migration, differentiation, mitogenesis, angiogenesis, embryogenesis, and wound healing [51,52]. FGF signaling plays a crucial role in both embryonic development and adult tissue homeostasis [53].

Depending on the cellular context, the activation of *FGFR* triggers three main downstream pathways, such as: the RAS/MAPK pathway, initiated via the FRS2 adaptor protein complex, which regulates cell proliferation and differentiation; the PI3K/AKT pathway, which controls cell survival and fate determination; and lastly, the PLCγ pathway, influencing cell morphology, migration, and adhesion [51,52].

Disruptions in FGF/FGFR signaling, including pathogenic genetic variants, can lead to aberrant skeletal development. These disruptions are associated with various types of skeletal dysplasia such as chondrodysplasia and craniosynostosis syndromes [54].

Among FGFR family members, *FGFR3* plays a pivotal role in the negative regulation of bone growth. Gain-of-function mutations in *FGFR3*—such as the well-characterized G380R variant—lead to excessive inhibition of endochondral ossification, resulting in conditions such as hypochondroplasia, achondroplasia (ACH), and thanatophoric dysplasia types I and II (TD I/II) [51,53].

Variants in *FGFR1* have also been associated with craniosynostosis and, in some reports, with short stature and other skeletal anomalies [55,56,57]. Although no direct association between *FGFR4* mutations and height has been reported, this receptor plays a role in cell fate decisions during embryonic muscle development and has been linked to hypertension and height variation in population studies [53,58].

The *FGFRL1* gene—recently recognized as the fifth member of the FGFR family—lacks intrinsic kinase activity but is implicated in cardiovascular and skeletal development. Variants in *FGFRL1* have been associated with congenital heart defects, skeletal malformations, bone mineral density alterations, height differences, and type 2 diabetes [59,60,61,62].

○ The Growth Hormone (GH)-Insuline- like growth factor-I (IGF-I) axis

The GH–IGF-I axis is essential for promoting linear growth during childhood and maintaining metabolic homeostasis in adulthood [63]. Beginning around the age of one, the GH–IGF-I system becomes the primary endocrine driver of postnatal height growth [64,65]. While, during prenatal development, *IGF-I* and *IGF-II* play critical roles in intrauterine growth. Both ligands activate the *IGF1R*, stimulating the PI3K/AKT signaling pathway, which is central to cell proliferation and tissue growth [65].

Genetic variants in key components of the GH–IGF-I axis—including *GH1*, *GHR*, *STAT5B*, *IGF1*, *IGFALS*, and *IGF1R*—can impair hormone signaling and lead to growth disorders such as GH insensitivity and short stature [66].

Mutations in the *GH1* gene can lead to impaired GH production or function, resulting in GH deficiency and associated growth failure [66]. More than 70 pathogenic variants have been identified in the *GHR* gene, with phenotypic effects ranging from mild to severe growth retardation [66]. GH binding to *GHR* activates the JAK-STAT signaling pathway; thus, *STAT5B* variants can result in severe postnatal growth failure, IGF-I deficiency, and GH insensitivity [66,67,68].

Mutations in *IGF1* and *IGF1R* can impair both intrauterine and postnatal growth, often manifesting as significant growth restriction [69]. Finally, defects in the *IGFALS* gene lead to acid-labile subunit deficiency, which reduces the stability of circulating *IGF-I* and *IGFBP-3*, thereby compromising *IGF-I* bioavailability despite normal or elevated hormone levels [66].

○ Wnt/β-Catenin Signaling

The Wnt/β-Catenin signaling pathway plays a crucial role in embryonic development and organogenesis, as well as in the regulation of growth and differentiation in various tissues. In the postnatal and adult skeleton, this pathway is essential for bone formation and maintenance [69].

The *CTNNB1* gene encodes the β-Catenin protein, which functions as a transcriptional co-activator [70]. Variants in *CTNNB1* can disrupt normal cellular processes such as proliferation, differentiation, and morphogenesis. Pathogenic variants have been associated with neurodevelopmental disorders that present with intrauterine growth restriction, followed by poor postnatal weight gain and short stature [71].

Moreover, the co-receptor *LRP5* is also a key component of Wnt signaling [72]. Variants in *LRP5* have been linked to disorders such as osteoporosis-pseudoglioma syndrome and high bone mass syndromes, both of which are associated with altered adult stature [73].

In addition, the *WNT1* gene encodes a Wnt ligand that, following translation and post-translational modification, activates the canonical Wnt/β-Catenin pathway [74]. Genetic variants in *WNT1* have been identified in individuals with osteogenesis imperfecta, a condition characterized by reduced bone mass and average height below the population mean [75].

○ Hedgehog signaling pathway

The Hedgehog (Hh) signaling pathway plays a key role in regulating cell proliferation, differentiation, and tissue patterning during embryonic development [76].

Disruptions in normal Hh signaling are associated with several human skeletal disorders. Genetic variants in key pathway components, including *IHH*, *PTCH1*, and *HHIP*—have been reported to influence human height by modulating growth plate development and endochondral ossification [77,78].

○ BMP/TGF-β Pathway

Bone morphometric proteins (BMPs) and transforming growth factor-β (TGF-β) are cytokines that play crucial roles in embryonic development, growth and differentiation. They are fundamental for skeletal development and maintenance of bone homeostasis [79,80].

BMP/TGF-β signaling can also activate non-canonical pathways such as p38 MAPK [80]. Furthermore, the cellular outcomes of this pathway are modulated through crosstalk with other key developmental signaling network, including Wnt, Hedgehog, *FGF*, Notch, as well as through extracellular antagonists, intracellular inhibitors, receptors ubiquitination and epigenetic modulation [80].

Several genes within this pathway have been implicated in height determination. For instance, *GDF5* (Growth Differentiation Factor 5), a member of the TGF-β superfamily, is expressed in the early stage of skeletal development and is involved in joint and cartilage formation [81]. Another gene, *BMPR1B* (Bone Morphogenetic Protein Receptor Type 1B), encodes a receptor that mediates BMP signaling and is essential for bone and limb development [82].

**Table 1:** Important pathway-genes and effects on height.

Gene	Pathway	Correlated with Height	Mechanism	Reference
*FGFR3*	FGF signaling	Gain of function variants are associated with short stature.	Inhibits chondrocyte proliferation in growth plate	[51,53]
*FGFR1*	FGF signaling	Limb and nervous system development	Short stature and variants in the gene are rare events.	[55,56,57]
*FGFR4*	FGF signaling	rs351855 and rs4752570 are associated with height	Effect on embryonic development	[53,58]
*FGFRL1*	FGF signaling	Height variation	Modulates cardiovascular system and bone formation	[59,60,61,62]
*GH1*	GH-IGF-I axis	Variants are linked to sever short stature. Other SNPs are normal height variants.	*GH1* encodes pituitary growth hormone.	[66]
*GHR*	GH-IGF-I axis	Variants identified in Laron syndrome and severe to mild growth failure	*GHR* mediates GH signaling in liver and growth plate; receptor defects impair JAK2–STAT5B activation	[66,68]
*STAT5B*	GH-IGF-I axis	Variants are linked to growth failure, IGF-I deficiency, and GH insensitivity.	Role in GH, essential for *IGF-1* transcription	[66,67,68]
*IGF1 and IGF1R*	GH-IGF-I axis	Mutations have benn associated with intra and post- natal growth retardation	Effect on the GH-IGF-I axis	[66]
*IGFALS*	GH-IGF-I axis	Variants result in growth failure.	Stabilizes the IGF-I -IGFBP3 complex, prolonging IGD-I half-life.	[66]
*CTNNB1*	Wnt/β-Catenin signaling	Neurodevelopmental disorders resulting in postnatal short stature	Encodes for the β-Catenin protein.	[70,71]
*LPR5*	Wnt/β-Catenin signaling	Osteoporosis-pseudoglioma and high-bone-mass syndromes and short stature	Wnt transduction in the signaling pathway	[72,73]
*WNT1*	Wnt/β-Catenin signaling	Osteogeneses imperfecta which cause short height.	Activates the signaling pathway.	[74,75]
*IHH*	Hedgehog signaling	Height associated	Role in endochondral ossification, major Hh input.	[77,78]
*PTCH1*	Hedgehog signaling	Height associated	Inhibits Smo and regulates Hh activation threshold.	[77,78]
*HHIP*	Hedgehog signaling	Height associated	Encodes for negative regulator of the Hh signaling pathway.	[77,78]
*GDF5*	BMP/TGF-β	Variants have been associated with symphalangism, brachydactyly, skeletal dysplasia and reduced stature	Encodes a protein that is member of the BMP ligand.	[81]
*BMPR1B*	BMP/TGF-β	Variants have been associated with symphalangism, brachydactyly and short stature	Encodes a protein that is member of the BMP ligand involved in cartilage formation	[81]

### 3.3. Growth-Related Genetic Variants in South American Population

While the majority of the genetic discoveries related to height have been derived from studies in European population, research in South American cohorts remains limited. However, emerging studies have begun to identify genetic variants that contribute to differences in stature, reflecting population-specific variants shaped by unique ancestral backgrounds and environmental influences.

One of the most notable findings in South America was reported by Asgari et al. (2020) [83], who studied Peruvians who are among the shortest people in the world. Their research identified a missense variant (E1297G) in the *FBN1* gene that was associated with reduced stature. Individuals carrying this variant were, on average, 2.3 cm shorter than non-carriers [83].

Additional evidence comes from consortia and GWASs. The Hispanic/Latino Anthropometry Consortium identified several SNPs associated with height in Hispanic/Latino population. Among these, a variant in *B4GALNT3* represented a novel signal not previously reported in global height studies [84]. Furthermore, a GWAS involving individuals of Hispanic ethnicity reported a total of 12, 111 independent SNPs significantly associated with height [50].

These findings underscore the importance of including underrepresented populations in genetic studies, as they may reveal ancestry-specific variants and mechanisms influencing human growth.

## 4. Clinical Growth Patterns in South American Children

For several decades, there has been a debate in South America regarding the most appropriate reference standards for establishing nutritional and short stature diagnoses. The discussion focuses on the ethnic origin of the individuals studied in the curves considered the international reference, as these studies do not provide an ethnic representation of the diversity of mixed races that exists in each South American country [85,86].

Additionally, the age at which pubertal development milestones (thelarche, pubarche, testicular enlargement, penile growth, pubertal growth spurt, menarche) occur varies in each population, reflecting genetic, environmental, and social factors. These different rates of pubertal development influence final height, so it would be expected that global reference curves for child growth should allow for correlation of height growth velocity with the Tanner stages, which is not possible with the current WHO international references [86,87].

There are two main types of growth charts reflecting distinct realities. Prescriptive growth charts follow individuals over time (longitudinal studies), usually for years. These charts show ideal growth in healthy individuals with optimal nutritional intake, access to all basic services, immunizations, and timely health checkups. The best example of this is the WHO growth chart (for children under 2 years of age), the product of a multicenter study, which aims to be a global reference for how breastfed children should grow, with optimal living and health conditions [5]. On the other hand, descriptive growth curves express the anthropometric characteristics of a population at the time of the study (cross-sectional studies). Examples of these curves exist in almost all countries in the region: Brazil [88], Argentina [85], Colombia [89], Venezuela [87], and Ecuador [8,90,91].

A longitudinal study of 232 urban Peruvian infants (aged 0–12 months), evaluated using WHO growth references. The results showed that growth trajectories were consistent with international standards. Prevalences of stunting, underweight, and wasting were all below 4%. Mean Z-scores for weight-for-age (0.17 ± 0.90 SD), length-for-age (0.07 ± 1.02 SD), and weight-for-length (0.18 ± 1.00 SD) remained close to zero, in sharp contrast to the 26.7% stunting prevalence reported in the 2000 Peruvian national survey. The authors attributed this advantage to prenatal iron and folic acid supplementation, along with the medical care provided during the trial. However, an increase in stunting prevalence was noted toward the end of the first year of life. Additionally, the researchers proposed that high-altitude ancestry was associated with greater length and chest circumference, possibly reflecting genetic adaptations involving the HIF-1 pathway [92].

A review of national surveys from thirteen Latin American countries further highlighted ethnic disparities in childhood stunting. In eight countries, Native American children had significantly lower height-for-age than the WHO growth reference group, even after adjusting for wealth and place of residence. While breastfeeding rates were higher in this group, complementary feeding practices showed lower dietary diversity. By contrast, children of African descent showed minimal differences—or, in some cases, lower stunting prevalence—compared with the reference group. These findings reinforce the need to prioritize Native American populations in child nutrition and development strategies to achieve the equity objectives outlined in the Sustainable Development Goals (SDGs) [93].

Similarly, a study conducted in Colombia, Bolivia, Guatemala, and Peru reported that children identified as Native American or afro-descendant were more likely to experience stunting or low weight. These findings were consistent even after adjusting to factors such as parental education level, socioeconomic status, and health. The study highlights the role of the social and educational environment on child growth patterns and recommends that interventions consider ethnic and racial minority groups [94]. Another study in Brazil found that growth estimates from the Food and Nutrition Surveillance System (SISVAN), which used WHO curves, tend to be higher than those recorded in national surveys such as National Demography and Health Survey (PNDS) 2006 or National Survey on Child Nutrition (ENANI) 2019. These results are because it focuses on the most vulnerable populations served by the public health system. This difference was attributed to the fact that SISVAN concentrates its surveillance on children served by the public primary care network, a group with greater nutritional vulnerability and less socioeconomic diversity [95].

In a study of the Guatemalan population, multiple national surveys (DHS/ENSMI) conducted between 1995 and 2014 were analyzed using the WHO 2006 international child growth standard. The results revealed inequalities associated with ethnicity, with higher prevalences of stunting in the Native American population [96]. Another study in the Mexican population assessed the nutritional status of children under five years of age using only WHO growth curves, classifying them as underweight, stunted, and wasted. The results showed a high prevalence of chronic malnutrition, with a greater impact in rural areas and among children from low-income households, as well as marked differences according to maternal educational level [97].

Ecuador currently uses growth curves established by the WHO; however, a national study identified discrepancies between Ecuadorian data and WHO reports. Specifically, the prevalence of low weight, short stature, and obesity appears to be underestimated or misclassified when WHO growth curves are applied. Likewise, several Latin American studies have shown that WHO curves detect higher prevalence rates of nutritional disorders—such as underweight, overweight, or obesity—compared with local references or older standards, including those developed by the Centers for Disease Control and Prevention (CDC) [98].

Recent data from Latin America highlights a significant discrepancy between international and local growth standards, resulting in considerable misclassification. In Ecuador, the use of the 2007 WHO growth charts identified 67.2% of schoolchildren as underweight, while the national reference classified only 7.5% in that category. The difference is even more pronounced for severe underweight, with 14.6% according to the WHO charts compared to just 1.1% using the national standards [98]. Conversely, obesity was underestimated (7.1% vs. 17.5%), and the agreement between systems was weak (Kappa ≤ 0.35), indicating that many children were categorized differently depending on the chart used [98]. Comparable inconsistencies were observed in Mexico, where the WHO criteria yielded higher prevalences of thinness (12.5% vs. 5.5% with CDC) and overweight (30% vs. 24.7% with IOTF) across more than 1700 Indigenous and non-Indigenous children [99,100]. Evidence from Peru further illustrates this issue: national birthweight-for-gestational-age curves identified 10.1% of neonates as small-for-Gestational-Age (SGA), whereas the Lubchenco standards detected only 4.1%, effectively missing more than half of vulnerable newborns [100]. Collectively, these studies suggest that anywhere from one in ten to more than six in ten children in the region may be reclassified depending on the growth standard applied, underscoring the diagnostic uncertainty introduced when global charts are used in heterogeneous Latin American populations.

These findings highlight the necessity of adopting growth monitoring approaches that consider both biological diversity and socio-environmental factors. Improving the methodological consistency of surveys is crucial for allowing more accurate comparisons between countries and specific interventions.

### 4.1. Growth Hormone Therapy and Precision Growth Medicine: Genetic Modulators of Response

#### 4.1.1. GH Therapy Indications

Growth hormone therapy is indicated for a range of pediatric conditions. For non-classical pathologies, the optimal timing for treatment initiation requires a more precise definition to maximize therapeutic benefit [101,102]. Although long-term follow-up generally demonstrates the safety of this therapy, potential risks include insulin resistance with compensatory hyperinsulinemia accelerated bone maturation with early onset of puberty, and changes in body composition [103]. Therefore, it is essential to rule out underlying clinical or genetic conditions that could limit treatment response and unnecessarily expose the patient to long-term risks [15]. In Prader–Willi syndrome, evidence supports GH therapy in children, adolescents, and adults, with reported benefits including increased height, improved body composition, and reduced BMI [104]. Similarly, approximately 10% of children born with SGA fail to achieve compensatory growth and remain short in adulthood. Clinical guidelines recommend initiating GH therapy between ages 2 and 4 to optimize growth acceleration; however, in practice, treatment often begins between the ages of 7 and 9, which reduces its potential benefits [105]. In idiopathic short stature (ISS), retrospective analyses indicate that rhGH therapy can produce an average height gain of 8–10 cm compared with untreated controls. Greater improvements are observed in non-familial cases, and the therapy maintains a favorable safety profile [106].

#### 4.1.2. Genetic Predictors of GH Response

**Figure 1:**
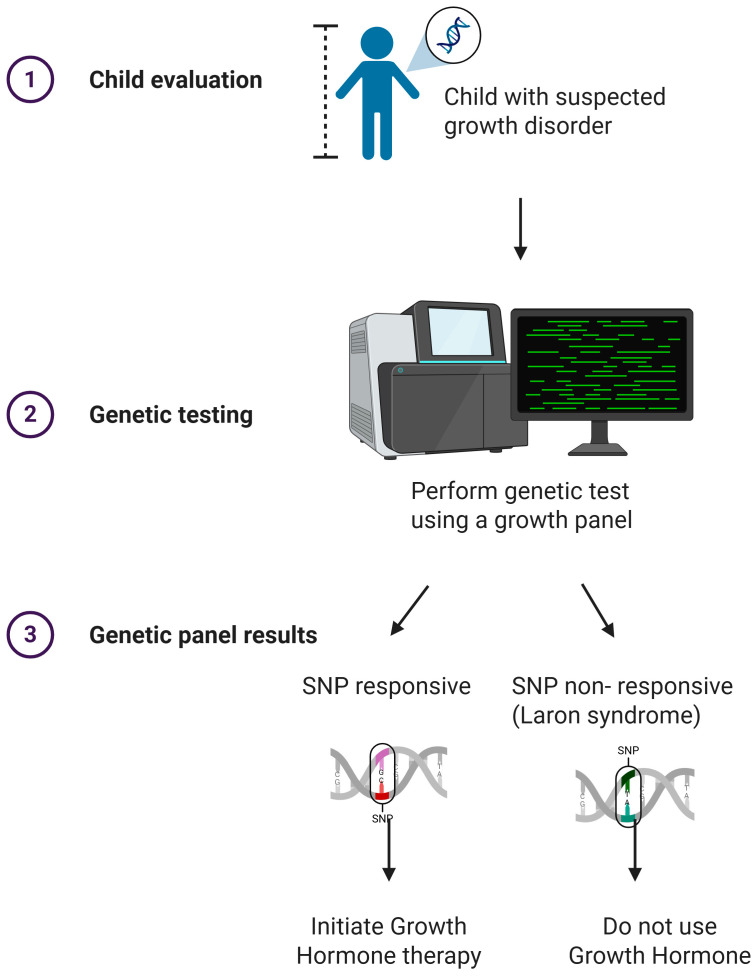
Suggested genetic considerations for GH treatment administration. Created in https://BioRender.com (accessed on 1 August 2025).

GH therapy remains fundamental in pediatric endocrinology, but interindividual variability in response has highlighted the importance of genetic predictors [107,108,109]. Current evidence shows that these markers differ in their level of validation, ranging from robust findings supported by meta-analyses to emerging associations from small cohorts or case studies [14,110,111] (Figure 1).

The most robust evidence comes from the deletion of exon 3 of *GHR* (d3-*GHR*), supported by a meta-analysis of 15 cohorts [112]. Yet, effect sizes vary across ancestries, suggesting limited generalizability. In Latin America, a retrospective cohort from Brazil confirmed improved growth velocity and final height in d3-GHR carriers [113]. Likewise, *SHOX* variants were also supported by an exploratory review of 22 studies [114] and validated in a Chilean cohort [115]. Both analyses consistently show significant improvements in growth velocity under GH therapy. *PTPN11* and *NF1* show therapeutic benefits, but with caveats: *PTPN11* carriers may experience skeletal complications [116], and NF1 patients need long-term safety monitoring due to the risk of tumors [117]. These finding underscores promise but also the need for replication in other admixed South American populations.

By contrast, several predictors remain emerging or uncertain. Variants in *ACAN* and *NPR2*, both associated with idiopathic short stature, exhibit favorable growth responses in small cohorts, although the results vary with the age at treatment initiation [118,119,120,121]. *IGF1*/*IGF1R* alterations are associated with partial or reduced responses, particularly in children born small for gestational age [122]. In Latin America, families with acid-labile subunit (ALS) deficiency have been reported with novel pathogenic variants, expanding the regional clinical-genetic spectrum and documenting partial responses [123]. A multicenter study spanning 12 countries in the region found that high treatment adherence, monitored using the digital device easy pod, resulted in an additional height gain of up to 1.1 cm over the first 24 months [124].

However, not all genetic growth disorders respond to rhGH. In Laron syndrome, GH resistance makes conventional rhGH therapy ineffective, as the defect resides in the GH receptor or its downstream signaling pathway, ultimately impairing the production of insulin-like growth factor 1 (*IGF-1*) [125,126]. As a result, patients typically present with normal or elevated GH levels but persistently low IGF-1 concentrations, leading to pronounced short stature. In these cases, the standard of care is the administration of recombinant human IGF-1 (rhIGF-1), which bypasses the defective GH signaling pathway and acts directly on the target tissues, thereby promoting linear growth and improving anthropometric outcomes [125,126].

Globally, evidence supporting this therapy is derived from meta-analyses and multicenter cohorts. In Latin America, however, reports are limited to small or single-center studies in Brazil [113], Chile [115], and Colombia [127]. This disparity underscores the urgent need for multicenter research that integrates the region’s genetic diversity into clinical practice.

While pharmacogenetics holds promise for refining GH therapy, its application should be considered with caution in resource-limited settings, where barriers to molecular testing and treatment access persist. Ensuring equitable access, improving adherence, and generating robust local data remain priorities.

Table 2 summarizes these findings, contrasting robust predictors such as *GHR* exon 3 deletion and *SHOX* with emerging candidates including *ACAN, NPR2*, and *IGF1/IGF1R*, and highlighting the disparity between global and Latin American evidence.

## 5. Environmental and Socioeconomic Modifiers of Growth

**Figure 2:**
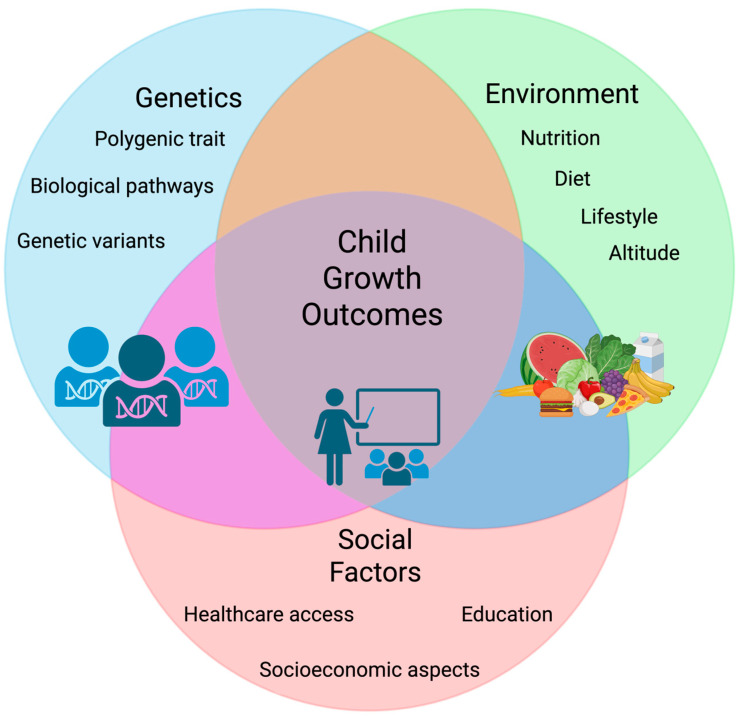
Integrative framework of child growth determinants in South America. Schematic representation of the interplay between genetics, environment, and social factors in shaping child growth outcomes. The intersection of these domains highlights how child growth results from the dynamic interaction of biological, environmental, and social contexts. Created in https://BioRender.com (accessed on 1 August 2025).

Child growth results from a complex interplay between biological, environmental, and socioeconomic factors (Figure 2), and it does not follow a strictly linear trajectory [135].

Growth is typically divided into three phases—infantile, childhood, and pubertal—each influenced by distinct determinants. Environmental and socioeconomic conditions, such as diet quality, access to healthcare, parental education, and exposure to infections, also play a decisive role in growth outcomes [135,136].

Adequate nutrition is a critical determinant of healthy development [136]. Malnutrition, whether caused by inadequate food supply or by underlying disease, can disrupt glucoregulatory, hormonal, and metabolic pathways, with long-term consequences for health and development [136].

Chronic child undernutrition remains a major public health concern in South America, where deep socioeconomic disparities persist. In contrast, in North America, childhood malnutrition more frequently manifests as overweight and obesity. However, in both regions, socioeconomic inequalities exacerbate these problems. For example, in the United States, children from low-income households and racial or ethnic minorities are more likely to experience food insecurity, which can lead both to insufficient nutrient intake and to obesity due to reliance on inexpensive, calorie-dense foods [137].

In South America, socioeconomic inequality continues to be a decisive factor in childhood nutritional status, with diverse manifestations of malnutrition. In Ecuador, recent studies have shown that maternal education, household income, and access to healthcare services are strong predictors of chronic undernutrition in children under five years of age [138]. Poverty and unfavorable living conditions, particularly in rural areas and marginalized communities, limit access to adequate food and basic services essential for healthy growth [139,140]. According to the Panorama of Food and Nutritional Security 2022 report by the United Nations, 22.5% of people in Latin America and the Caribbean lack sufficient resources to access a healthy diet. This figure rises to 52% in the Caribbean, 27.8% in Mesoamerica, and 18.4% in South America [141].

South America faces a dual burden of malnutrition, with undernutrition coexisting alongside overweight and obesity, intensified by socioeconomic disparities. A 2023 report revealed that millions of people in Latin America and the Caribbean cannot afford a healthy diet, with low-income households being disproportionately affected [142]. In Ecuador, the prevalence of chronic undernutrition is significantly higher among Native American populations. This inequity is further exacerbated in households with unmet basic needs, where the likelihood of chronic undernutrition in children under five increases considerably, underscoring poverty and ethnicity as key determinants of child growth [142].

Native American communities in the Andean and Amazonian regions of South America continue to exhibit disproportionately high rates of chronic child undernutrition, reflecting deep socioeconomic and environmental inequities. In countries such as Ecuador, the prevalence of chronic undernutrition among Native American children is significantly higher than the national average, exceeding other population groups by more than 10 percentage points [142].

Pediatric populations living in favelas, informal settlements, and marginalized urban neighborhoods in large South American cities may have relatively greater access to basic services compared to rural communities. However, precarious housing, inadequate sanitation, exposure to violence, and limited access to diverse and nutritious foods contribute to a dual burden of malnutrition. This includes persistent undernutrition in some children, alongside an alarming rise in overweight and obesity, driven by the widespread availability of ultra-processed foods and obesogenic urban environments [143].

Micronutrient deficiencies remain highly prevalent in Latin America. Iron deficiency is common and can lead to anemia, impairing motor and cognitive development. Zinc deficiency compromises immune function and increases susceptibility to infections, while vitamin A deficiency impairs vision and immune defenses. These deficiencies often coexist, reflecting diets lacking in diversity and nutrient density [144,145].

From a global perspective, WHO considers anemia a major public health issue, affecting more than 1.62 billion people, or 24.8% of the world’s population. The highest prevalence occurs among preschool-aged children. Each year, nearly 500,000 children under five die, with 27% of these deaths attributable to infectious diseases and malnutrition. Ensuring a well-balanced diet is therefore fundamental to human health. Nutrition serves not only as a critical indicator for assessing population health but also as a cornerstone for designing and implementing programs aimed at eradicating child malnutrition and improving quality of life across populations [144,145].

### 5.1. Infectious Disease Burden

The burden of infections further compounds the challenges to healthy growth in children. Low-income communities, particularly in rural or peri-urban areas, often lack adequate sanitation, safe drinking water, and proper waste management systems. These deficiencies contribute to a high prevalence of acute gastrointestinal and respiratory infections, which significantly increase the risk of undernutrition and stunting in children under five years of age [146].

Intestinal parasitism remains widespread in Latin America, with prevalence rates reaching up to 90% in certain study areas. Between 20% and 30% of the general population is infected with soil-transmitted parasites, but rates can climb to 50% in impoverished neighborhoods and up to 95% in some Native American communities [145,146]. In Peru, one study reported a 61.5% prevalence of intestinal parasitosis among children aged 6–12 years [147]. In Argentina, a multi-province study found infection rates exceeding 60% among children, with notable regional variation: Misiones province recorded the highest prevalence (82.0%), while Chubut reported the lowest (38.4%) [148]. In Venezuela, prevalence in individuals aged 2–18 years was 56.5%, with nearly half of cases in school-aged children. In a rural community in Monagas state, the prevalence reached 92.2% among children aged 0–15 years [149]. In Ecuador, rates vary by location, from 23.52% in rural Paute to 30.59% in Jipijapa [150].

Environmental exposures further exacerbate health risks. Air pollution, both indoor and outdoor, contributes to the respiratory disease burden among South American children. Fine particulate matter, gaseous pollutants, and biomass smoke from wood used for cooking or heating increase susceptibility to acute respiratory infections (ARIs), which can impair growth and development [151]. Similarly, water contamination, particularly with Escherichia coli, hinders progress in reducing chronic undernutrition, especially in rural and Amazonian provinces of Ecuador, Colombia, and Brazil. Limited access to safe drinking water and adequate sanitation infrastructure is therefore a critical environmental determinant of childhood nutrition in the region [152].

### 5.2. Gene–Environment Interactions in Child Growth

Child growth arises from complex interactions between genetic factors and environmental exposures, including nutrition (particularly protein and micronutrients), infection burden, altitude, and psychosocial stress [153]. Notably, genetics explains a fraction of height or growth-related traits; however, there is “missing heritability”, which may be mediated through environmental modulation of gene expression and epigenetic mechanisms [153,154].

In the South American Andes, high-altitude hypoxia exemplifies gene–environment interaction. Andean populations, such as the Aymara and Quechua, have lived for millennia in low-oxygen environments and show genetic signatures of positive selection, particularly, the nitric oxide pathway and cardiovascular system [155]. These adaptations are accompanied by physiological traits such as decreased hypoxic pulmonary vasoconstrictor response, and increased lung volume with enhanced cardiac O^2^ utilization, suggesting greater efficiency of oxygen use [156]. Environmental hypoxia, especially during pregnancy, also affects fetal growth, with epigenetic modifications likely mediating effects on placental development, oxygen transport, and later postnatal growth [157,158].

Nutritional deficits in early development also shape growth through epigenetic programming. Processes such as DNA methylation, histone modifications, and regulation by non-coding RNAs (e.g., microRNAs) alter the expression of growth-related genes without changing the DNA sequence [154]. Maternal nutrition and environmental exposures during the first 1000 days of life are particularly critical, with poor diet quality strongly associated with growth impairments and increased risk of obesity later in life [159,160].

Taken together, these examples underscore that in South America, understanding child growth requires not only identifying genetic variants (e.g., through GWAS) but also measuring environmental exposures, developmental timing (e.g., prenatal period and first 1000 days), and epigenetic or transcriptomic mediators. This integrative approach is crucial for identifying how environmental factors interact with genetic predispositions.

## 6. Limitations

A persistent limitation in the study of child growth in South America is the marked underrepresentation of Native American and Afro-descendant populations in genetic and genomic research [161]. Most GWASs have been conducted in cohorts of European ancestry, with minimal inclusion of admixed Latin American populations [162]. This lack of representation limits the accuracy of polygenic scores and reduces the predictive value of genotype–phenotype associations for South American children. Consequently, risk prediction, diagnostic thresholds, and therapeutic recommendations derived from European-centric data may be misleading when applied to diverse local populations.

Another important limitation is the continued use of growth charts developed from non-representative populations. Although WHO standards provide a valuable global benchmark, they are largely based on children from settings with different genetic backgrounds, environmental conditions, and socioeconomic contexts. This mismatch can lead to both over- and underdiagnosis of growth disorders, such as short stature, underweight, overweight, or obesity, in South American children [163]. Notably, some studies in Latin America have compared WHO standards and INTERGROWTH-21 for fetal growth assessment. The results showed that WHO standards can identify SGA neonates, but INTERGROWTH-21 showed better overall diagnostic performance [164]. Therefore, genetic differences do not always translate into clinically significant growth deviations. However, these findings should be interpreted in light of context- and region-specific factors that may influence growth. Beyond genetic considerations, socioeconomic inequalities remain a major determinant of child growth and quality of life in South America. High rates of poverty, unequal access to healthcare, inadequate sanitation, food insecurity, and low maternal education continue to disproportionately affect marginalized communities, particularly in rural, Amazonian, and high-altitude Andean regions. These conditions not only increase the prevalence of chronic undernutrition and micronutrient deficiencies but also exacerbate the burden of infectious diseases, creating a cycle of poor growth and compromised health outcomes.

## 7. Future Directions

1. Addressing these limitations requires a combination of immediate and long-term strategies that prioritize both biomedical and structural determinants of growth. The following areas emerge as the most actionable: Tackling malnutrition and its structural drivers: Public health policies must reduce socioeconomic inequities while integrating nutrition into healthcare delivery. Key priorities include reducing stunting, anemia in women of reproductive age, and low birth weight; preventing increases in childhood obesity; promoting breastfeeding; and decreasing child wasting [140]. The WHO also emphasizes the need for supportive environments, integration of nutrition into health interventions, adequate resource allocation, and systematic evaluation of intervention effectiveness.
2. Expanding ethnically inclusive genomic research: Given the overwhelming reliance on European-derived evidence, large-scale initiatives such as the Latin American Genomics Consortium (LAGC), JAGUAR, and the Genetics of Latin American Diversity (GLAD) project are critical. These efforts aim to create population-specific databases and atlases, uncover genetic diversity, and identify disease-relevant markers. Importantly, increased inclusion of Native American and Afro-descendant populations is essential to improve predictive models, reduce bias in diagnostics, and ensure equitable clinical translation of genomic advances [159]. Developing region-specific growth references. Growth standards tailored to the genetic, nutritional, and environmental contexts of South American subpopulations would improve diagnostic precision and reduce misclassification. While WHO charts will continue to serve as a global benchmark, complementary regional charts could support improved assessments, better identification of nutritional deficiencies, and more targeted interventions.
3. Integrating pharmacogenetics into clinical practice: Validated markers such as d3-GHR and SHOX can guide growth hormone therapy, while emerging candidates (e.g., ACAN, NPR2) require replication in admixed populations before routine use.
4. Improving treatment adherence monitoring: Expanding the use of digital devices (e.g., easy pod) to monitor adherence in GH therapy can optimize height outcomes and reduce variability in treatment response.
5. Ensuring equity in healthcare access: Policies must guarantee that underserved populations—particularly Native American and rural communities—have access to diagnostics, growth monitoring, and therapy, aligning with the Sustainable Development Goals (SDGs).

In summary, although all areas are crucial, immediate gains are most likely to be achieved by prioritizing malnutrition and public health inequities, given their widespread and direct impact on child growth and survival. In parallel, advancing inclusive genomics and refining growth references will provide longer-term benefits by enhancing diagnostic accuracy, risk prediction, and therapeutic equity across South American populations [159,163].

## 8. Conclusions

Child growth in South America results from a dynamic interplay of genetic, environmental, and socioeconomic factors, all influenced by the region’s unique ancestral diversity. Reliance on universal growth standards, while practical, carries the risks of misclassification and inappropriate clinical decisions when applied to populations whose growth trajectories differ from those represented in the reference data. Advances in genomics, nutrigenomics, and epigenetics now offer the opportunity to refine growth assessment, predict therapeutic responses, and design interventions that are both biologically and contextually relevant.

To fully realize this potential in South America, two priorities are essential: developing inclusive, population-specific growth standards that reflect the region’s diversity and implementing of pharmacogenomic-informed GH therapy to optimize treatment outcomes. Achieving these goals requires sustained regional collaboration, equitable access to molecular diagnostics, and the integration of genomic data with strong public health policies.

Ultimately, aligning growth assessment and therapy with South America’s unique biological and social realities will improve diagnostic accuracy, treatment efficacy, and equity, ensuring that all children in the region can achieve their full growth and developmental potential.

## Figures and Tables

**Table 2:** Summary of genetic predictors of response to GH therapy, contrasting global versus Latin American evidence.

Gene/Variant	Global Evidence	Latin American Evidence
*GHR* exon 3 deletion	Study: Wassenaar et al., 2009 (meta-analysis of 15 studies) [112]. N: meta-analysis of 15 studies Duration: 1 year Countries: Germany, Spain, The Netherlands, Taiwan, others Association: Carriers of d3 allele (*GHRd3*) showed higher baseline height SDS (+0.16), greater growth velocity (+0.52 cm/year), and modest height SDS gain (+0.075) vs. fl/fl. Effect stronger at lower GH doses and older age.	Study: Jorge et al., 2005 (retrospective cohort) [113]. N: 75 GHD children Duration: mean 7.5 ± 3.0 years Country: Brazil Association: d3 carriers had significantly higher growth velocity (12.3 ± 2.6 vs. 10.6 ± 2.3 cm/year) and taller final height (SDS −0.8 vs. −1.7; *p* < 0.05) than fl/fl homozygotes.
*SHOX*	Study: Sodero et al., 2024 (scoping review of 22 studies) [114]. N: 22 studies Duration: variable Countries: Germany, Italy, others Association: GH therapy improves growth velocity and is generally safe, though prospective studies are still required.	Study: Griffero González et al., 2024 (retrospective cohort) [115]. N: 9 *SHOX* patients (within 73 treated) Duration: ≥12 months Country: Chile (Santiago) Association: Significant height SDS gain (+0.8 ± 0.7; *p* = 0.007). Treatment well tolerated; adverse events mainly mild (headache, limb pain).
*NF1*	Study: Howell et al., 1998 (KIGS database, retrospective cohort) [117]. N: 102 GH-deficient NF1 children Treatment duration: up to 3 years Countries: International (Europe, USA) Association: Median height velocity increased from 4.2 cm/year (pre-treatment) to 7.1 cm/year in the first year and remained >5.7 cm/year at years 2–3. Median height SDS improved from −2.4 to −1.8 after 3 years. No excess malignant risk was detected compared with NF1 background incidence.	Not reported.
	Study: Haas-Lude et al., 2000 (retrospective, Germany) [128]. N: 10 NF1 patients Duration: variable, retrospective follow-up Countries: Germany Association: Overall, GH therapy was beneficial; one case showed tumor progression, another resolution. No second tumors or cutaneous neurofibromas were detected.	
	Case report: Vurallı et al., 2016 (NF1-Noonan syndrome) [129]. N: 1 girl (mutation in *NF1*) Duration: GH until final height Country: Turkey/France collaboration Association: Short stature due to GHD improved under GH treatment.	
*PTPN11*	Study: Jorge et al., 2022 [130]. N: 69 NS patients (71% *PTPN11*+) Treatment duration: 4 years of rhGH Countries: Multinational (Europe, USA, Japan) Association: Both *PTPN11*-positive and negative patients showed significant improvement in HSDS (+1.3 vs. +1.5 over 4 years, respectively; no statistical difference). Safety outcomes were consistent with prior GH studies.	Not reported.
	Study: Wu et al., 2023 (case series, China) [116]. N: 8 children with NS (7 treated with rhGH, *PTPN11*-positive) Treatment duration: median follow-up ≈ 3 years Country: China Association: Growth velocity increased from 3.7 ± 0.5 cm/year to 8.0 ± 1.0 cm/year (*p* < 0.01). One patient developed osteochondroma during therapy, highlighting the need for bone monitoring in *PTPN11* carriers.	
*IGF1/IGF1R*	Study: Çelik et al., 2022 (case report) [122]. N: 1 boy with complete *IGF1R* deletion Duration: 5.7 years of rhGH (two courses) Country: Turkey/The Netherlands Association: Improved growth velocity and near final height. Partial hypogonadotropic hypogonadism and central hypothyroidism developed. rhGH had partial effect; early initiation may be more beneficial.	Not reported.
	Study: Göpel & Pfäffle, 2021 (retrospective cohort) [131]. N: 23 *IGF1R* mutation carriers vs. 34 SGA controls Duration: ≥4 years rhGH Country: Germany Association: *IGF1R* carriers had lower growth response to rhGH (Δ height SDS 0.29 in year 1 vs. 0.65 in SGA, *p* < 0.01). Long-term NFH gain was modest (−2.59 SDS treated vs. −2.22 SDS in treated SGA).	
	Study: Zaitoon et al., 2024 (observational retrospective) [132]. N: 135 pediatric patients (64 GHD, 71 ISS) Duration: routine follow-up with BIA, cumulative rhGH dose assessed Country: Israel Association: GHD patients showed higher BMI z-scores, higher fat %, lower muscle-to-fat ratio compared to ISS. Higher IGF1 z-scores were positively associated with skeletal muscle mass but not with adiposity. Suggests rhGH therapy may mitigate muscle deficits by raising *IGF1*.	
*ACAN* variants	Study: Stavber et al., 2025 (comparative cohort, Slovenia) [133]. N: 17 children with *ACAN* variants (vs. 16 with *NPR2*) Duration: mean 5.3 ± 2.2 years of rhGH Country: Slovenia Association: Prepubertal start produced greater gains (+1.35 SDS) vs. pubertal (+0.3 SDS). *ACAN* group showed stronger overall response compared with *NPR2*.	Not reported
	Study: Sun et al., 2022 (familial short stature, China) [121]. N: 7 families (32 screened; 7 novel *ACAN* variants identified; 6 patients followed) Duration: mean 1.85 ± 1.91 years of rhGH Country: China Association: Height SDS improved from −2.89 ± 0.68 to −1.91 ± 0.93 after treatment. All showed good therapeutic response, expanding the pathogenic variant spectrum.	
*NPR2* variants	Study: Stavber et al., 2025 (comparative cohort) [133]. N: 16 children with *NPR2* variants (vs. 17 *ACAN*) Duration: mean 3.2 ± 1.7 years of rhGH Country: Slovenia Association: Prepubertal start yielded greater benefit (+1.01 SDS) than pubertal (+0.37 SDS). Effect positive but smaller than *ACAN* group.	Not reported
	Study: Chen et al., 2023 (case series) [134]. N: 3 unrelated Chinese patients with novel *NPR2* variants Duration: 2 years of rhGH Country: China Association: Height gain of +1.59 ± 0.1 SDS after 2 years. Functional studies confirmed severe loss of cGMP signaling in pathogenic variants.	

Abbreviations: GH, growth hormone; rhGH, recombinant human growth hormone; GHD, growth hormone deficiency; HSDS, height standard deviation score; GV, growth velocity; SGA, small for gestational age; ISS, idiopathic short stature; NFH, near final height; NF1, neurofibromatosis type 1; NS, Noonan syndrome; IGF1R, insulin-like growth factor 1 receptor.
